# Kidnapping-Induced Trauma and secondary stress in armed conflicts: a comparative study among women in hostage families, volunteers, and the General Population

**DOI:** 10.1186/s13584-024-00650-8

**Published:** 2024-11-04

**Authors:** Shahar Livne, Ilana Feldblum, Sara Kivity, Naama Shamir-Stein, Einat Brand, Shir Cohen, Eran Rotman, Hagai Levine, Mor Saban

**Affiliations:** 1 Health Policy and Management Department, School of Public Health, The Faculty of Health Sciences, Ben-Gurion University of the Negev, Beer-Sheva, 8410501 Israel; 2 Maccabi Healthcare Services, Tel Aviv-Jaffa, 6812509 Israel; 3 Braun School of Public Health and Community Medicine, Hadassah Medical Center, The Faculty of Medicine, Hebrew University of Jerusalem, Ein Kerem Campus, POB 12272, Jerusalem, 9110202 Israel; 4 Nursing Department, School of Health Professions, Faculty of Medical and Health Sciences, Tel Aviv University, Tel Aviv, Ramat Aviv, 69978 Israel

## Abstract

**Background:**

Exposure to armed conflict negatively impacts health. However, there is limited data on secondary stress from ambiguous loss contexts, such as kidnapping. In this study we aimed to quantify changes in modifiable health behaviors and well-being among women in hostage families and hostage crisis volunteers versus the general female population within the first two months of the 2023 Israel-Hamas war.

**Methods:**

A cross-sectional online survey was conducted on 318 Hebrew-speaking women aged 18–75 in Israel comparing: (1) a general population sample (*n* = 245); (2) hostage crisis volunteers (*n* = 40); and (3) hostage family members (*n* = 33). Participants provided demographic information, details on chronic illnesses, and responded to Likert-scale questions covering self-rated health, mental health, and lifestyle habits before the conflict and in current state.

**Results:**

Hostage family members reported the most severe health impacts, followed by volunteers. Fair/poor physical health status increased significantly in all groups during the war, with hostages’ families reporting the highest rate (61.6%). Mental health deterioration was more pronounced among hostages’ families, with 84% expressing a need for mental health support. Hostages’ families also reported the highest rates of sleep problems, reduced adherence to a healthy lifestyle, and weight loss. Mental and physical health declined significantly across the exposed groups, as measured by multiple assessments, with hostage families experienced the most pronounced impairments across various domains of well-being.

**Conclusions:**

This period of conflict severely harmed the well-being of all women in the study population. Women from all three groups - hostage families, volunteers, and those from the general population - experienced health deterioration due to varying levels of stress and exposure to conflict-related factors. Hostage families faced the greatest impact with nearly all members of this group showing significant health damage. Long-term support is needed to help restore post-conflict health for all affected women. Further research may be needed to determine the most effective interventions for addressing these impacts across the different groups.

## Key points

This was the first study to quantify changes in modifiable health behaviors and well-being among women indirectly exposed to conflict through a hostage situation.

Hostage family members experienced the most severe declines in mental health and other outcomes within 2 months of conflict onset compared to volunteers or the general population.

Conflict can have extensive negative impacts on community health through diverse experiences, even if individuals are not directly facing violence.

Tailored, long-term public health support interventions are needed to restore health in post-conflict periods, considering individuals’ varied burdens and exposure profiles.

## Introduction

Kidnappings that are inflicted during armed conflicts generate immense psychological trauma that reverberates beyond the direct victims [1, 2]. For example, research has begun to document the potentially severe psychological toll on relatives of those forcibly taken, including prolonged distress, the stress of uncertainty, and an inability to properly grieve [3]. While studies to date have primarily focused on abductions for financial ransom or prisoners of war (POWs), there is a critical gap in the literature regarding the effects of civilian abductions in the context of war and geopolitical tensions, particularly on their close relatives [4–6].

Months or years of living in anguish and grappling with the uncertainty of missing relatives can lead to what is known as ‘ambiguous loss’, a concept supported by a substantial body of evidence. Studies have documented the psychological impact of such loss on individuals and families [6, 7], and how it can trigger a distressing cycle of immense emotional strain, anxiety, and isolation [8, 9]. Moreover, families of abducted individuals may disproportionately endure additional traumatic stress due to direct or indirect exposure to the kidnapping event. This includes distressing visual material and testimonies used by both the kidnappers and their own side for identification or to assist in the situation [10]. Such exposure often leads to depression, anxiety, and complications of grieving indefinitely [9, 11]. Even volunteer organization members who are supporting close relatives of hostages may experience trauma due to their involvement in such dire circumstances and a paucity of resources available to them [12, 13]. Without adequate trauma support, preexisting or emerging disorders may develop under chronic strain [14–16].

Few studies have rigorously examined the health impacts of (i) populations directly dealing with the abduction of their relatives, and (ii) individuals volunteering to assist in such circumstances. However, these studies have tended to focus mainly on the long-term mental health effects on hostages’ families. In the case of armed conflicts, research typically focuses on the health of former abductees, mostly POWs, and their close families in the period following the abduction [17, 18].

The armed conflict between Israel and the Palestinian militant group Hamas started on October 7th, 2023, and has resulted in mass casualties and extensive destruction across the region [19]. The surprise assault by Hamas and other militant groups in southern Israel led to the abduction of over 250 individuals, including both Israeli and foreign nationals, who were forcefully taken to the Gaza Strip [20].

Diplomatic negotiations in November 2023 facilitated the release of 105 of the abducted individuals. Additionally, 4 persons were released in October 2023, and 8 were rescued in different Israeli Defense Forces (IDF) operations between October 2023 and August 2024. The bodies of 36 hostages were recovered by the IDF, while a further 35 hostages were declared dead with their bodies still held by Hamas at the time of writing. Despite diplomatic and military actions, as of September 2024, the fate of the remaining 66 hostages remains uncertain, leaving their families and communities without information as to their conditions or whereabouts [20, 21]. According to testimonies from those released in the hostages-prisoners exchange deal in November 2023, as collected by the Special Representative of the Secretary-General on Sexual Violence in Conflict, they were held in unbearable conditions, subjected to violence, including sexual assaults, starvation, forced dehydration, and lack of medical care [7]. Therefore, there is reasonable ground to believe that the remaining hostages are also suffering similar conditions. Nevertheless, the absence of confirmed details regarding their health, treatment, or potential release timelines, exacerbates the suffering of their relatives. Indeed, this uncertainty places them in a state of psychological limbo, locked in a parallel timeframe where their relatives are perceived as simultaneously living and dead [22, 23].

In response to this uncertainty, civil and non-governmental organizations (NGOs) have emerged to represent and support the hostages and their families. One of the main initiatives can be seen in the establishment of the “Hostages and Missing – Families Forum” (Forum), formed by hostages’ relatives and volunteers. The Forum provides support in various forms, including financial, legal, emotional, and medical assistance to the families. Additionally, it engages in advocacy with local government ministries and international bodies, supporting the goal of the safe return of the hostages. Volunteers at the Forum, particularly within the first three months of the war, dedicated themselves daily to providing support to the families and continue to do so, absorbing their heart-wrenching emotions, and listening to their painful stories.^1^ As mentioned earlier, these volunteers themselves may potentially suffer secondary stress.

The ongoing armed conflicts highlight the fact that there is currently insufficient research quantifying the cascading costs for these uniquely exposed and often-marginalized hostage families and hostage crisis volunteers. Understanding their experiences, needs, and responses over time is crucial to their advocacy and to designing interventions alleviating preventable post-conflict suffering. This study therefore aimed to investigate the mental and physical health impacts on Israeli families of relatives kidnapped and the volunteers who assisted them in comparison to the general population. The study’s insights may inform targeted public health support and policy to better serve vulnerable hostage families, aid workers, and communities affected by abductions.

## Methods

### Study population and sampling methods

This was a cross-sectional study comparing three distinct population groups (described below) conducted in November-December 2023 during the initial months of the Israel-Hamas war. The study sample included Hebrew-speaking Israeli citizens aged 18–75 years who were active members of any of the four health maintenance organizations (HMOs) in Israel. Participants were screened for eligibility based on the sampling framework:

1) A representative sample of 245 women was drawn from the general population using stratified random sampling. To ensure a balanced and unbiased sample, the random sampling process monitored key demographic factors including HMO affiliation, age, and geographic region. Data from this group was collected via an online questionnaire which was administered by a professional survey company (iPanel) in November 2023.

2) A convenience and snowball sample of 40 female volunteers who provided assistance to hostages’ families through the Hostages and Missing Families Forum during the study period.

3) A convenience and snowball sample of 33 female family members of abductees during the study period.

Data collection for both the volunteer and family groups was carried out by the Forum through the administration of an online questionnaire in December 2023. Although the survey was distributed to both men and women, the higher response rate from female participants from the latter two population groups led us to focus our analysis on women. The inclusion of these three distinct groups – the general population, the families undergoing the trauma of having kidnapped relatives, and the volunteers supporting them – enabled a comparative analysis of the physical and mental health impacts experienced during this conflict.

### Ethics approval and consent to participate

This study adheres to the principles of the Declaration of Helsinki. Approval was received from the Tel-Aviv University Institutional Review Board prior to the commencement of the study (0007724-1). The survey was distributed to the general population through an established research panel (https://www.ipanel.co.il/en/) where participants already consented beforehand to taking surveys. Additionally, the survey was distributed through the Forum, an organization that has gained the trust of both families and volunteers involved in the study. Careful consideration was given to the wording of questions in order to avoid causing unnecessary distress given the sensitive topic. The confidentiality and anonymity of responses were strictly maintained with no identifying information collected. The data was securely stored and was only reported in aggregate form.

### Data collection

Data collection for the general population sample was conducted by Maccabi Healthcare Services through the iPanel online survey platform. iPanel maintains a pool of Israeli respondents who have consented to periodically complete surveys on various topics [24]. From this pool, iPanel recruited a representative 245-person sample that reflected the Israeli population. The online questionnaire was distributed via the iPanel service infrastructure in late November 2023, approximately one month following the initiation of the conflict in October 2023. This timing allowed for assessing short-term public health impacts while events remained recent.

The same online questionnaire was distributed to family members of hostages and volunteers by the Forum. Due to the vulnerability and relatively small size of these two populations, achieving a random sample was not feasible. To protect the well-being of the participants, we collaborated closely with the Forum to invite participants through its family representatives and volunteer departments via WhatsApp and internal organizational channels. The online questionnaire was administered in December 2023, with invitations extended to eligible participants aged 18 and above of Israeli nationality. While this approach did not provide a random sample, it allowed us to reach these difficult-to-access populations while prioritizing their well-being. The survey was fully answered by 73 eligible participants, comprising 33 family members and 40 volunteers.

### Study tools

The evaluation of the respondents’ status was conducted through a questionnaire structured into three key domains: self-rated health (SRH), mental health, and lifestyle habits and behaviors. The SRH domain included two questions about the participants’ perceptions of their general health, each assessed on a 5-point Likert scale (ranging from poor to excellent). In the mental health domain, we asked 11 validated questions [25] assessing sleeping problems, emotional distress and depression symptoms, the need for therapy, and the use of medications for depression and sleep problems. Most questions employed a 5-point Likert scale, with questions on medication measured by a binary scale of yes or no. In the third domain, we asked questions regarding adherence to healthy nutrition, physical activity, preventive medicine, and smoking habits. This domain utilized both 5-point and 3-point scales for measurement. Additionally, participants were asked demographic questions, and about their chronic illnesses using a yes/no format for a checklist. Participants were requested to provide responses pertaining to their status prior to the conflict, their current state, and any discernible discrepancies between these two temporal points.

### Data analysis

Quantitative analysis was conducted using SPSS software Version 29, with statistical significance set at *p* < 0.05. Descriptive statistics provided an overview of response patterns across the general population, volunteer, and family samples. Response distributions between the three groups for Likert-scale questions were analyzed using chi-square tests to assess associations between categorical variables. These included examining impacts such as reported stress levels across groups. For analysis purposes, categories were combined from a 5-point Likert scale to 3-point Likert. Assumptions of expected cell frequencies were verified before conducting the chi-square tests. Bonferroni post-hoc tests were then used to explore the differences in response distributions between specific groups, with corrections made for multiple comparisons.

## Results

A total of 318 female participants were included, comprising 245 from the general population, 40 volunteers, and 33 hostages’ family members. Within the general population, 49.8% were aged 18–34, 34.7% were 35–54, and 15.5% were 55–75. This younger distribution was different from the volunteer group, where only 10% were aged 18–34 while 50% were in the oldest category of 55–75. The age profile of the hostage family group was more similar to the general population, with 39.4% aged 18–34 and 36.4% aged 35–54, which is still significantly higher than the volunteer group for the youngest category based on the Bonferroni post-hoc correction (*p* < 0.001).

### General health

As seen in Table 1, there were significant differences in general health status before and during the war between the three study groups. Before the war, a fair/poor general health status was reported by 11.8% of the general population, 5.1% of volunteers and 15.4% of hostages’ family members with no significant differences. However, during the war these percentages increased significantly, with 44% of volunteers and 61.6% of hostages’ families reporting fair/poor general health compared to 23.7% of the general population (*p* < 0.001 for both comparisons). The percentage of individuals who reported a deterioration in general health status from before to during the war was also significantly higher among volunteers (66%) and hostages’ families (77.9%) compared to the general population (40.4%, *p* < 0.001 for both comparisons).

**Table 1:** Comparison of general health outcomes among general population, volunteers, and hostage families: pre-war and during-war perspectives

Variable	General Population (a)	Volunteer Group (b)	Hostages Family Group (c)	*P*-value^12^
General Health Status – Before War (Fair/Poor) (n(%))	29 (11.8)	2 (5.1)	5 (15.4)	0.309
General Health Status – during War (Fair/Poor) (n(%))	58 (23.7)	18 (44)_a_	20 (61.6)_a_	< 0.001
Deteriorated Health Status^3^ (n(%))	99 (40.4)	26 (66)_a_	26 (77.9)_a_	< 0.001
Postponed/Given Up Medical Appointments or Tests (n(%))	70 (40)	19 (57.8)	25 (85.7)_ab_	< 0.001

^1^ Chi-square test; Bonferroni correction

^2^ Significance levels between groups are reported in the P-value column, with the significant group labeled by the corresponding letter (group a, b or c)

^3^ Calculated assessing the adverse shift in perceived health status from pre-war to war time

**Fig. 1:**
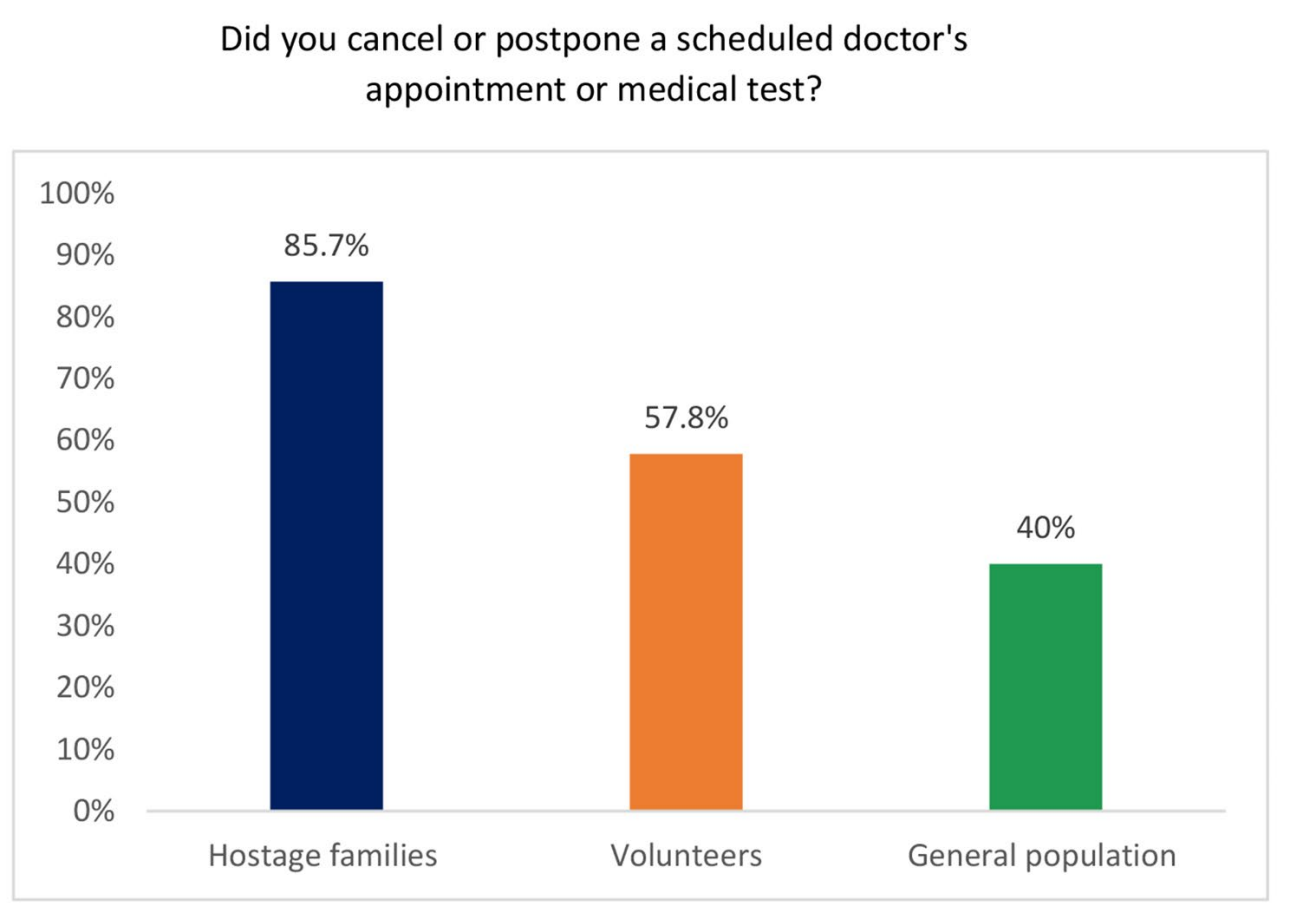
Disruptions to medical care access after war onset

As presented in Fig. 1, a higher percentage of volunteers (57.8%), and especially hostages’ family members (85.7%), reported postponing or giving up medical appointments/tests during the war compared to the general population (40%, *p* < 0.001 for both comparisons).

### Mental health

Similar patterns were observed for mental health status as shown in Table 2. Before the war, a fair/poor mental health status was reported by 13.9% of the general population and 19.7% of volunteers with no significant differences, while it was 0% among hostage family members. During the war, these percentages increased sharply, with 69.7% of volunteers and 67.3% of hostage families reporting fair/poor mental health compared to 40.8% of the general population (*p* = 0.001 for both comparisons). A higher percentage of volunteers (80.9%) and especially hostage families (96.1%) also reported a deterioration in mental health status from before to during the war compared to 60.8% of the general population (*p* < 0.001 for both comparisons). Similarly, reporting of constant/frequent emotional problems and seeking of psychological assistance during the war was significantly higher among volunteers and hostage families compared to the general population. There was no significant difference between the groups in the overall use of medications for anxiety and depression both before and during the war. However, the hostages’ family members group showed a notable increase in medication use, rising from 7.7 to 19.3%. In contrast, the general population and volunteer groups showed minimal changes.

**Table 2:** Comparison of mental health outcomes and status among general population, volunteers, and hostage families: pre-war and during-war perspectives

Variable	General Population (a)	Volunteer Group (b)	Hostages Family Group (c)	*P*-value^12^
Mental Health Status – Before War (Fair/Poor) (n(%))	34 (13.9)	7 (19.7)	0 (0)	0.027
Mental Health Status – during War (Fair/Poor) (n(%))	100 (40.8)	28 (69.7)_a_	22 (67.3)_a_	0.001
Deteriorated Mental Health Status^3^ (n(%))	149 (60.8)	32 (80.9)_a_	32 (96.1)_a_	< 0.001
Constant/Frequent Emotional Problems (n(%))	102 (41.6)	31 (77.7)_a_	25 (75)_a_	< 0.001
Seeking Psychological Assistance (n(%))	56 (22.9)	11 (27.1)	28 (83.6)_ab_	< 0.001
Using Medications for Depression/ Anxiety – Before War (n(%))	41 (16.3)	10 (25.6)	3 (7.7)	0.109
Using Medications for Depression/ Anxiety – during War (n(%))	39 (15.5)	10 (26.4)	7 (19.3)	0.158
Taking Prescription Sleeping Pills – Before War (n(%))	19 (7.8)	3 (7.3)	0 (0)	0.326
Taking Prescription Sleeping Pills – during War (n(%))	29 (11.8)	8 (20.5)	7 (21.2)	0.058

^1^ Chi-square test; Bonferroni correction

^2^ Significance levels between groups are reported in the P-value column, with the significant group labeled by the corresponding letter (group a, b or c)

^3^ Calculated assessing the adverse shift in perceived health status from pre-war to war time

### Sleep problems

Almost all respondents from hostage families reported a deterioration in the quality of their sleep. Following the war onset, 21.2% of the hostage family group reported taking prescription sleeping pills. As expected, the increase in the use of sleeping pills is higher among hostage families compared to women in the general population.

### Adherence to a healthy lifestyle

As shown in Table 3, compared to the pre-war period, adherence to a healthy lifestyle deteriorated significantly more among volunteers and especially hostage families during the war. Specifically, a higher percentage of hostage families (33.6%) reported no adherence to a healthy lifestyle during the war compared to 12.2% of the general population and 16.8% of volunteers (*p* = 0.001). Similarly, reporting of deterioration in nutritional habits and physical activity was also highest among hostage families.

**Table 3:** Comparison of adherence to healthy lifestyle among general population, volunteers, and hostage families: pre-war and during-war perspectives

Variable	General Population (a)	Volunteer Group (b)	Hostages Family Group (c)	*P*-value^12^
Adherence to a Healthy Lifestyle – Before War (None) (n(%))	14 (5.7)	0 (0)	2 (3.8)	0.034
Adherence to a Healthy Lifestyle – during War (None) (n(%))	30 (12.2)	7 (16.8)	11 (33.6)_a_	0.001
Deterioration in Nutritional Habits (n(%))	110 (44.9)	22 (55.9)	29 (87.5)_ab_	< 0.001
Significant Weight Loss (n(%))	4 (1.6)	1 (2.2)	7 (21.2)_ab_	< 0.001
Deterioration in Physical Activity (Complete Break) (n(%))	35 (17.8)	9 (24.2)	16 (56.9)_ab_	< 0.001

^1^ Chi-square test; Bonferroni correction

^2^ Significance levels between groups are reported in the P-value column, with the significant group labeled by the corresponding letter (group a, b or c)

### Weight changes

**Fig. 2:**
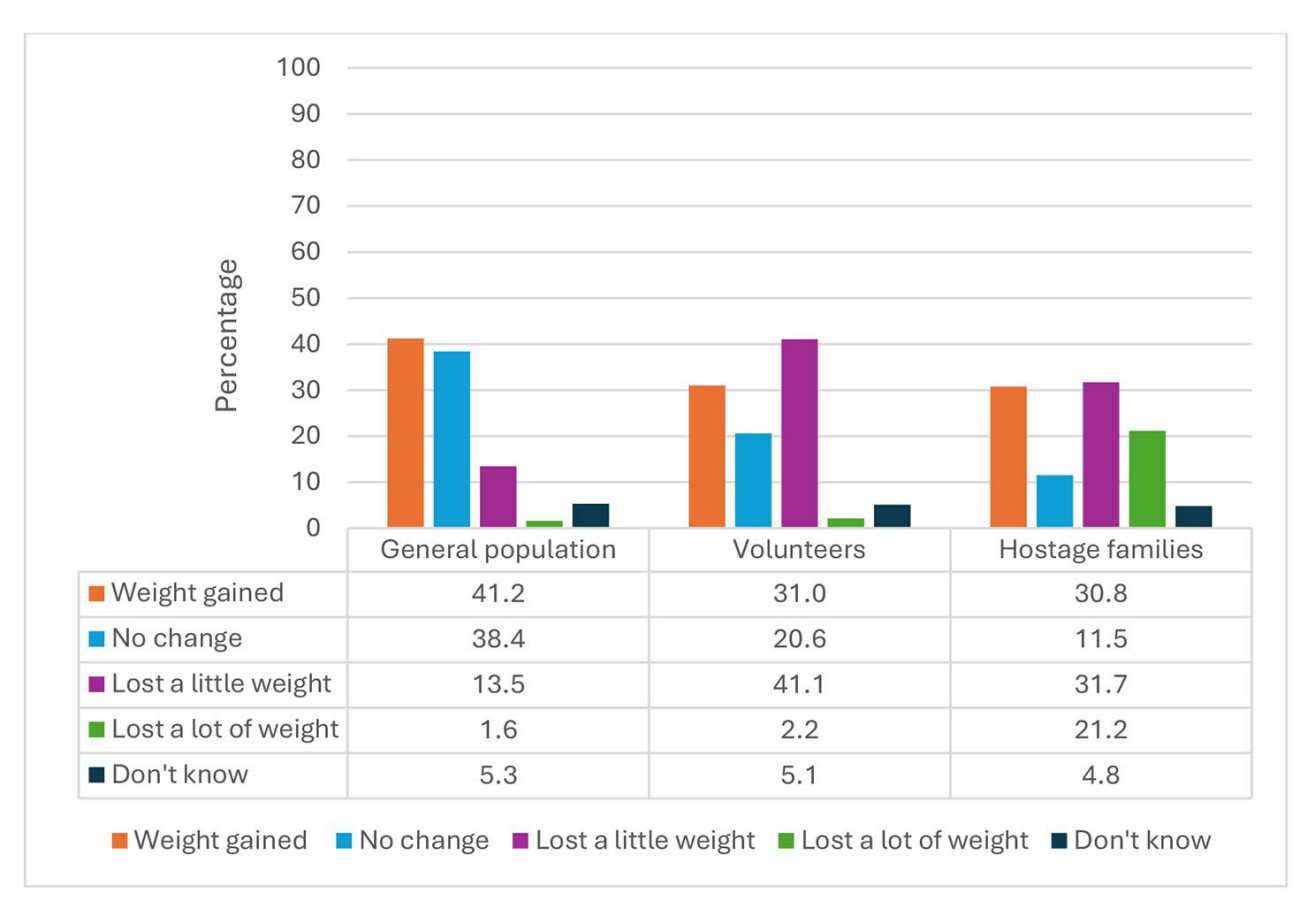
Weight changes during the war

Figure 2 illustrates weight changes experienced by women during the war period. It displays substantial differences between the study groups. Among the general female population, 38% reported maintaining their weight during the war months, while 15% lost weight and 41% gained weight. In contrast, among the family group, 32% lost some weight, and 21% lost a lot of weight. Considerably fewer managed to keep their weight stable (12%), while 31% gained weight. Volunteer women showed a different pattern between the other two groups. Weight loss was reported by 43% of this group. 21% maintained their weight, and 31% gained weight.

### Physical activity

Reports of decreased physical activity were found in all groups. In the hostage family group, in particular, over half stopped engaging in physical activity completely. This proportion was higher compared to volunteers and women in the general population.

## Discussion

The results of this study provide concrete statistical evidence regarding the immediate common health impacts of armed conflict on vulnerable populations, specifically hostages’ relatives and their supporters. By quantitatively assessing changes from pre-conflict baselines among hostage families, volunteers, and the general population, in a cross-sectional survey, we documented the pervasive and pronounced effects even months after violence began.

The findings illuminate how close involvement with hostage crises markedly elevates the risk of secondary trauma. Those with familial connections to victims, unsurprisingly, given the affective bonds to the primary sufferers, exhibited the most pronounced impairments compared to other groups, as supported by previous studies [26]. While our study does not directly measure uncertainty, the unique nature of the trauma experienced by these families, may be related to the burden on their health and well-being. Previous research has already shown that individuals facing kidnapping or detention of relatives endure heightened anxiety, helplessness, and grief [27, 28]. The concerns about a family member’s safety and well-being likely overwhelmed their normal coping capacities, a key component of resulting trauma. Our findings align with previous research, suggesting that urgent medical attention to mental and physical healthcare is imperative for such groups in crisis contexts. While our data do not specifically indicate that establishing routines is therapeutic, it is a common recommendation in crisis management [29].

The volunteer study group also reported substantial health impacts. Any resources the volunteers were able to draw on from mobilizing aid efforts did not fully protect them from experiencing negative health effects. This aligns with evidence suggesting that volunteer work carries risks of secondary traumatization and burnout without self-care [30, 31]. Indeed, the study’s volunteers are from the Forum and tirelessly support the families of hostages, exposing themselves to an emotional roller coaster of fears, and trauma. The volunteers are in close contact with families and are also exposed to testimonies, death notices of hostages, and visual materials. They are not protected from the ambiguous loss, which extends beyond the hostages’ families, emphasizing the necessity of ensuring mental health support for crisis responders as both an ethical duty and a facilitator of longer-term recovery [32, 33].

More broadly, this timely study provides empirical evidence that violence undermines population health far beyond injuries. Armed conflicts jeopardize not only physical safety but also the ability to maintain healthy lifestyles and manage chronic conditions; impairments that can propagate future vulnerability 34, 35, 36, 37]. The observed declines in exercise, nutrition, sleep quality, and healthcare adherence across the study’s subgroups also align with the theory that prolonged stress “gets under the skin” to influence disease risk via pathways like immune dysregulation and unhealthy behaviors [3].

These findings have important and immediate policy implications. Mitigating longer-term consequences will require addressing social and environmental adversity contributing to poor health, beyond the clinical treatment of resulting diseases such as weight gain and depression or anxiety disorders [38]. Population-level responses prioritizing mental wellness, health behaviors, and self-management skills during instability periods may help curb the upstream social determinants of morbidity [39]. Investing in community-level interventions across affected communities and populations with unique exposure to kidnapping – specifically families of the hostages – could contribute to strengthening support networks and, consequently, enhancing health status. The Israeli health system and different HMOs should proactively engage with relatives of hostages and other conflict-affected communities, offering support through preventive medicine, rescheduling missed appointments, suggesting mental health support, and more. Complementary qualitative research elucidating civilians’ experiences could further guide protective interventions.

While this study focused on the negative health impact of traumatic wartime kidnappings, further research could provide a more comprehensive understanding by examining potential coping strategies and protective factors, such as belief systems and support networks, that may help individuals to cope better with these situations. In addition, future studies should investigate longitudinal patterns of medical condition management and whether conflict exposure exacerbates pre-existing issues or precipitates new complications over extended periods. Additionally, the potential for enduring, far-reaching consequences on long-term health status remains unclear without follow-up evaluations. While initial weight changes could be assessed through this cross-sectional design, fully characterizing the complexity of health burdens imposed on especially vulnerable populations like hostage family members will demand a more comprehensive, long-term research approach attuned to diverse outcomes and trajectories over time. This study’s findings offer preliminary insights but emphasize the need for more rigorous analyses capturing the breadth and depth of conflict-related suffering across multiple health domains and temporal scales.

In conclusion, this study provides timely and compelling evidence that armed conflict jeopardizes health through multiple pathways that manifest rapidly and disproportionately impact vulnerable groups. While more research is still needed, the findings reinforce the necessity of conflict-sensitive health planning that anticipates population needs, strengthens resilience factors, and ensures continued access to core services during times of duress, with flexibility and adaptive capacity for changing needs. Coordinating multisectoral action between humanitarian, public health, governmental, and community stakeholders, including civil society, can help to proactively address both immediate health declines and their potential long-term consequences. For those enduring immense suffering due to exposure to the abduction of their relatives, prioritizing mental healthcare, health behaviors, social support systems, and disease management represents a meaningful form of aid that alleviates suffering in the present, while also safeguarding future wellness. Overall, the study emphasizes population health protection as a fundamental component of achieving care, recovery, and justice for civilians living through or beyond crisis contexts.

### Limitations

This study has several limitations. As a cross-sectional survey, we could only assess changes retrospectively from self-reports rather than longitudinally monitoring outcomes over time. Recall and reporting biases also pose limitations when retrospectively assessing pre-war mental health status. Relying on self-reported recall of past mental health is subject to inaccuracies over time as the literature shows that recall can diminish significantly. This recall bias may confound determining changes from pre- to post-war, as overly positive pre-war recollections are possible. Prospective designs or records could objectively measure baseline functioning and minimize recall bias [40] but was not feasible here since the abduction, due to its unpredictable nature, could not have been predicted. An additional limitation arises from the focus on women in the analysis, meaning we cannot draw conclusions about the impact of war and ambiguous loss on the health of males who are relatives of hostages and their male supporters. Previous research suggests that men are less likely to report anxiety, depression, and PTSD symptoms after traumatic exposure compared to women [41]. This potential gender difference could have implications for tailoring policy interventions and support strategies for this population. This study is also limited by the lack of closer age conformity between the three groups. Moreover, the two-month timeframe captures only initial changes, and long-term follow-up would be needed to understand persistence or recovery trends over prolonged unrest. Information on chronic conditions, pre-existing mental health diagnoses, and medication use was also limited. Unmeasured confounders like variations in exposure to violence or displacement could have differently influenced subgroups. Finally, cause-and-effect relationships between conflict exposure and observed outcomes cannot be determined from our observational study.

Despite these limitations, this study offers real-time and robust documentation of the effects of the current conflict, both on the general population and unique groups, specifically the hostages’ families. This could facilitate the development of tailored public health interventions to address current and relevant needs, ultimately leading to improved outcomes. However, further studies are needed to validate these interventions. Additionally, by focusing on the hostages’ family group, this research provides unique insights into the immediate health impacts on populations that were rarely studied prior to the release of their relatives, especially in the context of armed conflicts. This timely approach enhances the novelty and significance of the findings and contributes to our understanding of the effects of armed conflict on civilians, particularly those in close circles of hostages.

## Conclusion

This study provides robust quantitative evidence of the profound and multifaceted impacts of an armed conflict on health and well-being. Specifically, our findings illustrate how warfare can rapidly undermine social structures and individual resilience. Amid such instability, targeted support and population health protections are needed to counteract conflict-exacerbated harm and safeguard vulnerable groups. Building on these results, continued research can further strengthen our understanding of the acute effects of violence on non-combatant populations. With deeper insights, policymakers will be better equipped to design timely, nuanced interventions and reforms promoting community resilience in the face of adversity. Overall, the evidence emphasizes the importance of considering the far-reaching welfare consequences of conflict and informing humanitarian efforts accordingly.

## Data Availability

Data will be made available per reasonable request.

## Footnotes

1 For further information, please see the Forum website: https://stories.bringthemhomenow.net/about.
